# Knowledge, Attitude, and Barriers to Fluoride Application as a Preventive Measure among Oral Health Care Providers

**DOI:** 10.1155/2018/8908924

**Published:** 2018-04-16

**Authors:** Aqdar A. Akbar, Noura Al-Sumait, Hanan Al-Yahya, Mohammad Y. Sabti, Muawia A. Qudeimat

**Affiliations:** ^1^Department of General Dental Practice, Kuwait University, Kuwait City, Kuwait; ^2^Ministry of Health, Kuwait City, Kuwait; ^3^Department of Developmental and Preventive Sciences, Kuwait University, Kuwait City, Kuwait

## Abstract

**Objective:**

To investigate the knowledge, attitude, and possible barriers to fluoride application among oral health-care providers in Kuwait.

**Methods:**

A validated self-administered questionnaire was distributed to a random sample of 291 dentists. The questionnaire included four categories: dentists' characteristics, knowledge of and attitude towards fluoride application, factors influencing decision-making on prescription of fluoride, and the clinician's perception of own knowledge. Means, group differences, and logistic regression were calculated.

**Results:**

262 completed the questionnaire (response rate of 90%). Half of the participants (49%) reported that water fluoridation is the best method for caries prevention in children. Majority of the participants (80%) acknowledged that topical fluoride prevents dental caries, but only 40% frequently use it in their practices. Fear of overdose was a concern in 57% of the participants. About 31% believed that caries is a multifactorial disease and cannot be prevented. In addition, 32% of the dentists who thought caries is multifactorial and cannot be prevented stated that restorations take precedence over preventive therapy.

**Conclusion:**

Despite the participants being in favor of topical fluoride application and believing in its effectiveness, certain barriers were apparent such as knowledge deficiencies, products labelling flaw, and lack of participation in effective continuing educational activities.

## 1. Introduction

A major decline in the prevalence of dental caries has been observed over recent decades. This decline has been attributed to the widespread use of daily fluoride toothpaste [1]. Kuwait is considered a nonfluoridated community since water fluoridation was discontinued in 1980 [2]. In addition, salt, milk, and juice are not fluoridated, and individuals have access to both fluoridated and nonfluoridated toothpaste. Despite the preventative effort from the Ministry of Health and the School Oral Health programs, caries is still considered a major problem [2, 3]. Al-Mutawa et al. [3] found that only 24–32% of 4- and 5-year-old children were caries-free, and the decayed score of the dft/dfs was the major component of the mean scores. Similar scores were reported for DFT/DFS in 12- and 14-year-olds [4].

The sizable amount of available dental literatures in addition to frequent controversies among clinicians and researchers has made decision-making for dental care and treatment planning very complex [5, 6]. Decision-making on caries diagnosis and management is primarily based on factors related to the dentist's characteristics, knowledge and experience, and to patient and practice factors [5, 7]. Dentists' knowledge and attitudes toward evidence-based clinical practices are very important to the profession to be able to offer the best possible care to the patient and to effectively influence their oral health behavior [8–10].

The American Dental Association (ADA) and the National Institute of Health and Care Excellence (NICE) place emphasis on the prevention and early detection of dental caries as the most important elements in any health-care program [11, 12]. With the current level of evidence, fluoride is well documented as an effective preventative method against dental caries for people at risk of developing dental caries via enhancing remineralization and inhibiting demineralization [13–19]. The application of this knowledge in clinical practice seems deficient and not well adopted [9, 10, 20, 21]. Bansal et al., reported that dentists showed a lack of understanding of fluorides' main mechanism of action which could lead to inappropriate judgement on the effectiveness of its use in different age groups [20]. Another study demonstrated a positive attitude towards preventative dental care but a deficiency in the knowledge regarding the role of fluoride in caries prevention as well as underestimation of fluoridated toothpaste role in caries control and reduction [9]. Investigators concluded that many dentists are not prepared well neither to prescribe the right fluoride regimen nor to council patients/parents about the appropriate fluoride use [22]. The aim of this study was to assess the knowledge, attitude, and possible barriers to fluoride application among dentists in Kuwait.

## 2. Material and Methods

Ethical approval was granted from the Health Science Center Ethical Clearance Committee, Kuwait University, and the Ethics and Research Committee, Kuwait Ministry of Health. All participants provided a signed informed consent. The study was conducted in full accordance with ethical principles, including the Declaration of Helsinki. A questionnaire was designed to investigate knowledge and attitudes as well as barriers to fluoride use as a preventative measure among dentists in Kuwait. The questionnaire was developed according to previous surveys, ADA guidelines, and the most recent available evidence [9, 14, 16, 17, 21]. A pilot study was performed on ten dentists (later excluded from the final sample) working at the Faculty of Dentistry, Kuwait University. This was done to assure that the survey questions were well formulated relating to the objectives of the study and that questions are well understood by the targeted dentists. Face validity was measured against a construct definition. Twenty-four items received 10 out of 10, and five items received 6 out of 10 and were removed mainly because the dentists thought they were unrelated to the objectives of the study. The validated survey was then readministered to the same 10 dentists, and all 24 questions were correctly answered by all participants.

The questionnaire was divided into 4 sections and consisted of 24 questions. In the first section, the dentists reported on their demographics, dental training, and practice after graduation. The second and third sections investigated the dentists' knowledge and attitude towards fluoride and its application. The last section examined the dentists' perception of their own knowledge regarding fluoride applications and the best methods for obtaining new evidence-based information.

The final sample size was calculated based on a confidence level of 90% and marginal error of 5%. At the time of carrying out this study, there were 1160 registered general dentists and specialists working in Kuwait (as per the latest manpower statistics of the Kuwaiti Ministry of Health) [23]. Therefore, the required sample size was estimated at 219 participants. To account for a possible 25% drop out/refusal, 291 dentists were met in person and invited to participate in this study. The study population was randomly selected by a multistage random-sampling method.

A total of 291 dentists working in the six health districts of the country from primary care clinics, specialty care clinics, and the private sector were invited to participate and complete the self-administered questionnaire.

Data were coded, verified, and analyzed using SPSS (version 18; SPSS Inc., Chicago, IL, USA). The logistic regression and chi-square tests were used for the analysis. A probability level of less than 0.05 was considered as statistically significant.

## 3. Results

A total of 291 dentists were invited to participate, and 262 (176 males and 86 females) completed the questionnaire, giving a response rate of 90%.  Table 1 summarizes the participants' characteristics, in which specialty was further divided into three groups: general dental practitioner, specialist caring for children (including pediatric dentists, orthodontists, and dental public health), and a third group of other specialties.

The participants' perception of the most effective methods for caries prevention in children and adults is shown in Table 2. Almost half of the participants (49%) stated that the most effective methods for caries prevention were water fluoridation for children and fluoridated toothpaste for adults. In addition, a few of the dentists believed that caries cannot be prevented in both children and adults (4% and 8%, resp.).

Table 3 shows results of the logistic regression model for dentists who chose water fluoridation as the most effective fluoride regimen to prevent dental caries in children. Undergraduates from dental schools of Northern America region were found to be more in favor of water fluoridation than undergradutes from other regions.

Table 4 illustrates the participants' belief of topical fluoride benefits and risks. The majority believed that topical fluorides can prevent caries (80%), make enamel more resistant to caries attacks (95%), and that it is safe in recommended concentrations (91%).

When asked about the application of professional topical fluoride, 40% reported frequent use, 47% claimed using it occasionally, whereas 13% never used it. Fluoride gel was reported by 65% of participants to be the most frequently used form of professional topical fluoride application, followed by rinses (5%) and varnish (3%). In addition, 35% of the participants reported the correct application time of 4 minutes for the gel form. Reasons for not supporting topical fluoride application in clinical practice are listed in Table 5.

Table 6 shows results of logistic regression models for the odds of favoring restorative treatment over prevention, which was significantly higher for dentists who believed that caries is a multifactorial disease and cannot be prevented.

Table 7 shows results of logistic regression models for dentists who believed that caries is a multifactorial disease and cannot be prevented. Few dentists who graduated from Asian undergraduate programs agree that caries cannot be prevented.

When participants were asked about their perception of their own knowledge, 60% claimed that they have adequate knowledge regarding topical fluorides. The majority (69%) stated that they needed further information regarding topical fluorides. In addition, 63% of the total participants reported attending topical fluoride continuing education (CE) sessions in the past 5 years or less, and 45% of the participants reported that the best method to obtain new information is through special courses.

## 4. Discussion

Topical fluoride application in the form of toothpastes, mouth rinses, varnishes, and gels has been shown to prevent dental caries [13, 14, 16, 17]. In this questionnaire, when dentists were asked about the most effective methods of fluoride regimens to prevent dental caries for both children and adults, the responses varied. Surprisingly, 49% stated that for children, water fluoridation is the most effective method; whereas for adults, 49% reported fluoridated toothpaste as being the most effective. Only 11% thought that fluoridated toothpaste is more effective for children compared to other application forms. This suggests that for children, dentists believe that the main effect of fluoride is primarily during the preeruptive stage. Similar findings were also reported in a previous study, where only 5% of participants identified that the posteruptive effect of fluoride surpasses any preeruptive effects [20]. Yoder et al. [21] also found that the majority of dental professionals were unaware of the fluoride's predominant posteruptive mode of action. Understanding the mechanism of action of any therapeutic agent—in this case fluoride—is critical since it will help in providing the best preventive programs for the patient, which will eventually maximize disease control [13, 20, 21]. In addition, believing that water fluoridation is the most effective method of caries prevention in children may affect parents counseling and education of tooth brushing methods and frequency by causing them to underestimate the importance of these methods [20, 21, 24].

In the logistic regression model, dentists who graduated from a Northern American undergraduate dental program were in favor of water fluoridation as the most effective fluoride regimen to prevent dental caries in children. One explanation could be that undergraduate curricula from different universities were suggested to have an influence on dentists' knowledge as reported by different studies [25, 26]. Some authors found that most dentists depend on knowledge gained from their undergraduate studies as the main source of information for their daily practice [26]. Different clinical guidelines and protocols as well as clinical training can also contribute to such beliefs [25, 26]. It is also possible that some participants confused the terms “cost-effectiveness” and “most effective”, which might have affected their choice.

Most of the dentists in this study (80%) reported that topically applied fluoride has a beneficial effect in caries prevention for both children and adults. Almost 95% stated that professionally applied topical fluoride in the form of varnishes, gels, and foams makes enamel more caries resistant. Still, only 36% believed that it is more beneficial than systemic fluoridation, which clearly shows the confusion in fluoride's predominant mode of action. Even though 91% of participants believed that it is safe in recommended concentrations and application protocols, 57% still have fear of overdose. This developed fear may be due to that studies and trials rarely provide information on toxicity and adverse effects [14, 20].

When it comes to clinical application, 65% of the participants reported that topical fluoride in gel form was their preferable method of choice. However, 65% of those were unfortunately unaware of the optimal application time of the gel form and will use it for less than 4 minutes, which may minimize the overall effectiveness and benefits. Even though some of the manufacturers recommend an application time of only 1 minute, this duration of application was not supported by the literature [20]. Some studies suggested that flaws in product-labelling and manufacturer instructions may play a role in the dentist's ability to correctly use many of the available fluoride products which will affect their effective use and counseling with patients [21]. In other studies, the matter of labelling confusion was also raised for varnish products. Varnish products are FDA-approved to be used as cavity liners and not as a preventive agent [22]. The recommendation of using varnish to prevent caries is described as “off the label” [22]. This imprecise labelling of different topical fluoride agents may cause confusions and eventually barriers to its application. In addition, the handling properties of topical fluoride agents can play a role in its application. Participants reported using topical fluoride irregularly in their clinic, in which only 40% frequently apply it to their patients. Since the majority of our participants are using fluoride gel, it could be the handling properties of the gel that hinder their frequent use. As documented in the literature, fluoride gel is very effective as an anticaries agent; however, it has some drawbacks such as the bitter taste, as well as the 4-minute waiting experience with an ill-fitting tray, which can be an unpleasant experience [22].

In this era, with all the advancements in research, knowledge, and dental technologies, it was surprising to see that 31% of our participants did not support topical fluoride application for caries prevention because they believed that caries is a multifactorial disease and cannot be prevented. The acceptance of the classical term “multifactorial disease” could influence dentists' choice and affect their decision of adopting preventative measures in their routine dental practice.

A logistic regression model showed a significant association between dentists who believed that restorative treatment should take precedence over prevention and those who believed that caries is a multifactorial disease and cannot be prevented. Dental caries is frequently described as a multifactorial disease process [27, 28]. Recent reviews suggested that with broader understanding of the disease's process, we can consider the dietary sugars to be the main cause of the disease and the other factors as causal factors that speed the disease process [29, 30]. By understanding the disease's process in its broader definition, we can conclude that treatment of dental caries can be achieved through nonoperative procedures that include dietary and plaque control along with remineralization therapy [31, 32]. In addition, the philosophy of “drill and fill” to treat the disease in early stages could dictate the dentist's treatment decision-making, and the concept of minimally invasive dentistry is still facing some obstacles to its application by some dentists [8]. Moreover, undergraduate dental education from different universities was found to significantly play a role in the dentists' belief that caries is a multifactorial disease and cannot be prevented, to which few Asian undergraduates agreed. It could be that the Asian curriculum is more affiliated with the European system that has been described for many years to adopt a preventative treatment philosophy [33]. Also, differences in education had an effect on both preventative knowledge and preventative dental behaviors amongst Asians as reported by Soh [34].

Contradiction amongst our participants was evident when the majority (60%) claimed that they had adequate knowledge regarding topical fluorides but still 67% reported that they needed further information. In addition, 63% reported attending topical fluoride continuous education (CE) sessions in the past 5 years or less. Hence, there seems to be some doubts and uncertainties when it comes to the knowledge and use of fluoride.

In our study, 45% of the participants stated that the best method to obtain new information is through special courses, lectures and seminars; 23% through scientific journals; 17% through newsletter; and 15% through the World Wide Web. The literature shows that interactive educational meetings through attending workshops and participation in active discussions with the lecturers is the most effective intervention to diffuse certain knowledge and thus changing clinical practice [35]. However, distribution of passive educational material like guidelines and publications, didactic educational meetings, and lectures have little or no effect on changing practitioners' knowledge or attitude and eventually cause a change in their routine dental practice [35]. To overcome the deficient knowledge among dentists working in Kuwait, an annual interactive workshop that highlights the importance of dental caries prevention, effective strategies, and available materials is highly needed to influence the change and improve the current dental knowledge, attitude, and practice.

In conclusion, despite the belief in topical fluoride effectiveness, certain barriers were apparent to its application. Knowledge deficiencies and attitude of practitioners play a major role. Clinical uncertainty as a result of labelling flaws, outdated undergraduate education, inappropriate continuous education, and lack of participation in effective educational activities are barriers too, and they can hinder clinicians from practicing evidence-based dentistry in their routine dental practice.

## Figures and Tables

**Table 1:** Participants' characteristics.

Characteristics	*n* (%)
Sex	
Male	179 (67)
Female	86 (33)

Age	
≤30 years	107 (43)
31–45	114 (46)
≥46	27 (11)

Nationality	
Kuwaiti	147 (57)
Non-Kuwaiti	111 (43)

Region of undergraduate dental education	
North America	38 (16)
Europe	40 (16)
Asia	59 (24)
Middle East	109 (44)

Year of practice	
≥10	155 (61)
<10	101 (39)

Specialty	
General dental practitioners	147 (57)
Specialist (PD, ORTHO, and DPH)	39 (15)
Other specialists	71 (28)

Work place	
Primary care clinics	97 (38)
Specialty care clinics	120 (46)
Private clinics	42 (16)

Area of practice	
Rural	113 (44)
Urban	142 (56)

**Table 2:** Participants' beliefs regarding the most effective fluoride regimen to prevent dental caries in children and adults.

	Children *n* (%)	Adult *n* (%)
Water fluoridation	98 (49)	30 (15)
Fluoride toothpaste	23 (11)	101 (49)
Fluoride rinses	1 (1)	10 (5)
Professionally applied topical fluoride	61 (30)	44 (21)
Fluoride supplements (tablets and drops)	10 (5)	4 (2)
Caries cannot be prevented	8 (4)	17 (8)

**Table 3:** Logistic regression model for dentists who believed (dependent variable) that water fluoridation is the most effective fluoride regimen to prevent dental caries in children.

Variables	Systemic (%)	Topical (%)	Caries cannot be prevented (%)	Odds ratio	CI (95%)	*P*
Gender						
Males	55.1	39.0	5.9	0.26	0.46–0.98	0.48
Females (reference)	50.8	49.2	0.0	—	—	—

Age group (years)						
≤30	51.8	43.5	4.7	0.66	1.11–2.43	0.46
31–45	53.8	41.8	4.4	0.90	0.51–2.32	0.21
≥46 (reference)	55.6	44.4	0.0	—	—	—

Region of undergraduate dental education						
North America	71.0	29.0	0.0	1.55	2.58–0.52	0.003
Europe	63.3	33.3	3.3	0.90	1.88–0.07	0.07
Asia	54.5	43.2	2.3	0.66	1.67–0.35	0.20
Middle East (reference)	44.7	48.2	7.1	—	—	—

Specialty						
General dental practitioner	50.4	44.3	5.2	0.05	1.22–1.12	0.93
Specialists caring for children	62.1	37.9	0.0	0.53	1.54–0.49	0.31
Other specialists (reference)	55.6	40.7	3.7	—	—	—

Years of practice						
≥10	51.6	43.4	4.9	0.25	−0.81–1.31	0.65
<10 (reference)	58.1	39.2	2.7	—	—	—

Area of practice						
Rural	50.0	43.9	6.1	0.18	0.50–0.85	0.61
Urban (reference)	55.4	41.5	3.1	—	—	—

Work place						
Primary care clinics	51.2	47.5	1.2	0.25	0.91–1.42	0.67
Specialty care clinics	52.7	40.7	6.6	0.54	0.48–1.55	0.30
Private (reference)	65.5	34.5	0.0	—	—	—

Topically applied fluoride has no risk of overdosing						
Agree	54.8	38.7	6.5	0.50	1.81–0.81	0.45
Disagree	54.7	43.6	1.7	0.32	1.56–0.93	0.62
Not sure	47.4	42.1	10.5	—	—	—

**Table 4:** Participants' beliefs regarding benefits and risks of professional topical fluoride application.

	Agree *n* (%)	Disagree *n* (%)	Not sure *n* (%)
Can prevent caries	205 (80)	27 (10)	25 (10)
Has a beneficial effect on children's oral health	239 (93)	6 (2)	13 (5)
Has a beneficial effect on adults' oral health	183 (72)	37 (14)	36 (14)
Makes enamel more caries resistant	241 (95)	6 (2)	8 (3)
Is preferable to systemic fluoridation (water, tablets, or drops)	91 (36)	109 (43)	53 (21)
Is preferable to brushing twice a day with fluoride toothpaste	64 (25)	173 (68)	17 (7)
Decreases the interest in tooth brushing	31 (12)	199 (78)	25 (10)
Is safe in recommended concentration and application	230 (91)	9 (4)	14 (5)
Has no adverse effects	82 (32)	145 (57)	29 (11)

**Table 5:** Reasons for not applying professional topical fluoride application in clinical practice.

Factors	Agree *n* (%)	Disagree *n* (%)	Not sure *n* (%)
Restorative treatment should take precedence over prevention	40 (16)	186 (78)	13 (6)
Busy in practice, no time for topical fluoride application	44 (18)	186 (77)	13 (5)
Caries cannot be prevented since it is a multifactorial disease	75 (31)	145 (59)	24 (10)

**Table 6:** Logistic regression model for dentists who believed (dependent variable) that restorative therapy should take precedence over preventative therapy.

Variables	Agree (%)	Disagree (%)	Not sure (%)	Odds ratio	CI (95%)	*P*
Gender						
Males	16.5	78.7	4.9	−0.30	−1.13–0.53	0.48
Females (reference)	16.0	76.5	7.4	—	—	—

Age group (years)						
≤30	21.2	72.7	6.1	0.58	−1.19–2.35	0.52
31–45	12.0	85.2	2.8	0.05	−1.36–1.46	0.95
≥46 (reference)	16.7	75.0	8.3	—	—	—

Region of undergraduate dental education						
North America	11.1	88.9	0.0	0.67	−0.52–1.86	0.27
Europe	13.9	80.6	5.6	0.85	−0.32–2.02	0.16
Asia	1.8	94.6	3.6	1.28	−0.51–2.61	0.06
Middle East (reference)	26.0	68.3	5.8	—	—	—

Specialty						
General dental practitioners	21.2	72.3	6.6	−0.03	−1.31–1.26	0.97
Specialists caring for children	7.7	89.7	2.6	0.21	−1.01–1.43	0.74
Other specialists (reference)	12.3	84.6	3.1	—	—	—

Years of practice						
≥10	20.1	74.3	5.6	−0.66	−1.89–0.57	0.29
<10 (reference)	10.4	84.4	5.2	—	—	—

Area of practice						
Rural	14.3	78.6	7.1	0.06	−0.73–0.84	0.88
Urban (reference)	17.2	77.7	5.1	—	—	—

Work place						
Primary care clinics	19.8	74.7	5.5	−0.14	−1.48–1.22	0.85
Specialty care clinics	15.8	78.9	5.3	−0.30	−1.51–0.90	0.62
Private (reference)	10.5	84.2	5.3	—	—	—

Dental caries cannot be prevented because caries is a multifactorial disease						
Agree	32.4	58.1	9.5	−2.34	−3.94–0.73	0.004
Disagree	9.7	88.3	2.1	−1.16	−2.68–0.36	0.14
Not sure (reference)	8.3	75.0	16.7	—	—	—

**Table 7:** Logistic regression model for dentists who believed (dependent variable) that caries cannot be prevented because it is a multifactorial disease.

Variables	Agree (%)	Disagree (%)	Not sure (%)	Odds ratio	CI (95%)	*P*
Gender						
Males	30.7	58.3	11.0	0.23	0.89–0.43	0.49
Females (reference)	30.9	61.7	7.4	—	—	—

Age group (years)						
≤30	31.3	56.6	12.1	1.01	0.38–2.39	0.16
31–45	29.2	63.2	7.5	0.33	1.40–0.74	0.55
≥46 (reference)	32.0	56.0	12.0	—	—	—

Country of undergraduate dental education						
North America	19.4	75.0	5.6	0.67	0.24–1.57	0.15
Europe	22.9	68.6	8.6	0.88	0.02–1.77	0.06
Asia	14.3	73.2	12.5	1.05	0.10–2.01	0.03
Middle East (reference)	45.7	43.8	10.5	—	—	—

Specialty						
General dental practitioners	35.0	56.9	8.0	0.18	1.20–0.84	0.73
Specialists caring for children	17.9	71.8	10.3	0.54	0.34–1.43	0.23
Other specialists (reference)	31.2	57.8	10.9	—	—	—

Years of practice						
≥10	32.6	57.6	9.7	0.48	1.45–0.50	0.34
<10 (reference)	28.4	61.1	10.5	—	—	—

Area of practice						
Rural	39.3	52.4	8.3	0.42	1.04–0.20	0.18
Urban (reference)	26.3	62.8	10.9	—	—	—

Work place						
Primary care clinics	36.3	56.0	7.7	0.50	1.54–0.53	0.34
Specialty care clinics	30.1	57.5	12.4	0.59	1.50–0.32	0.20
Private (reference)	18.4	73.7	7.9	—	—	—

Restorative treatment should take precedence over prevention						
Agree	60.0	35.0	5.0	1.61	3.49–0.27	0.09
Disagree	22.8	67.7	9.5	0.05	1.82–1.72	0.96
Not sure	50.0	21.4	28.6	—	—	—
